# Isolation of Highly Pathogenic H5N1 Influenza Viruses in 2009–2013 in Vietnam

**DOI:** 10.3389/fmicb.2019.01411

**Published:** 2019-06-25

**Authors:** Gongxun Zhong, Shufang Fan, Tiago J. S. Lopes, Mai Quynh Le, Harm van Bakel, Jayeeta Dutta, Gavin J. D. Smith, Jayanthi Jayakumar, Hang Le Khanh Nguyen, Phuong Vu Mai Hoang, Peter Halfmann, Masato Hatta, Yvonne C. F. Su, Gabriele Neumann, Yoshihiro Kawaoka

**Affiliations:** ^1^Department of Pathobiological Sciences, School of Veterinary Medicine, University of Wisconsin–Madison, Madison, WI, United States; ^2^National Institute of Hygiene and Epidemiology, Hanoi, Vietnam; ^3^Department of Genetics and Genomic Sciences, Icahn School of Medicine at Mount Sinai, New York, NY, United States; ^4^Duke-NUS Medical School, Singapore, Singapore; ^5^Duke Global Health Institute, Duke University, Durham, NC, United States; ^6^Division of Virology, Department of Microbiology and Immunology, Institute of Medical Science, University of Tokyo, Tokyo, Japan

**Keywords:** influenza virus, H5N1, Vietnam, surveillance, deep-sequencing

## Abstract

Routine surveillance and surveillance in response to influenza outbreaks in avian species in Vietnam in 2009–2013 resulted in the isolation of numerous H5N1 influenza viruses of clades 1.1.2, 2.3.2.1a, 2.3.2.1b, 2.3.2.1c, and 2.3.4.1. Consistent with other studies, we found that viruses of clade 2.3.2.1c were dominant in Vietnam in 2013 and circulated in the northern, central, and southern parts of the country. Phylogenetic analysis revealed reassortment among viruses of clades 2.3.2.1a, 2.3.2.1b, and 2.3.2.1c; in contrast, no reassortment was detected between clade 2.3.2.1 viruses and viruses of clades 1.1.2 or 2.3.4.1, respectively. Deep-sequencing of 42 of the 53 isolated H5N1 viruses revealed viral subpopulations encoding variants that may affect virulence, host range, or sensitivity to antiviral compounds; virus isolates containing these subpopulations may have a higher potential to transmit and adapt to mammals. Among the viruses sequenced, a relatively high number of non-synonymous nucleotide polymorphisms was detected in a virus isolated from a barn swallow, possibly suggesting influenza virus adaption to this host.

## Introduction

Highly pathogenic avian influenza (HPAI) viruses of the H5N1 subtype were first identified in Vietnam in 2001 (Nguyen et al., 2005). Since 2003 Vietnam has reported more than 2,700 outbreaks of HPAI H5N1 in poultry, far more than any other country (Thailand and Egypt have experienced the second and the third largest numbers of HPAI H5N1 outbreaks in poultry with 1,141 and 1,084 reported events since 2003, respectively^1^). The large number of HPAI H5N1 outbreaks in Vietnamese poultry in 2003–2005 was paralleled by 93 laboratory-confirmed human infections with HPAI H5N1 influenza viruses in Vietnam, 42 of which were fatal. Since 2015 no human HPAI H5N1 infections have been reported in Vietnam, but HPAI H5N1 viruses remain a threat to the country’s public health.

Since their first emergence in 1997 in Hong Kong, HPAI H5N1 viruses have evolved into multiple clades and subclades that are defined on the basis of the sequence of the viral surface glycoprotein hemagglutinin (HA), the major viral antigen. In Vietnam, HPAI H5N1 viruses of multiple clades have been identified, including clades 0, 1, 2.3.2, 2.3.2.1, 2.3.4, 3, 5, 7, and subclades thereof (Influenza Research Database^2^). The major outbreaks in 2003–2005 were caused by viruses of clade 1, which circulated throughout the country (Wan et al., 2008; Le and Nguyen, 2014). No outbreaks were reported in 2006, presumably due to a vaccination campaign (Le and Nguyen, 2014). In 2007–2009, viruses of clade 1 were predominantly isolated in the southern part of the country, whereas clade 2.3.4 viruses of the Fujian lineage (and sub-clades thereof) were detected in the northern part of the country (Dung Nguyen et al., 2008; Le et al., 2008; Wan et al., 2008; Nguyen et al., 2012; Le and Nguyen, 2014). A national vaccination program with a clade 2.3.4 vaccine virus may have led to the eradication of clade 2.3.4.1, 2.3.4.2, and 2.3.4.3 viruses, which have not been reported since 2010 (Le and Nguyen, 2014). Instead, viruses of clade 2.3.2 became dominant in 2009–2010 in the northern part of the country and diversified into subclades 2.3.2.1a and 2.3.2.1b (Nguyen et al., 2012; Creanga et al., 2013; Okamatsu et al., 2013; Le and Nguyen, 2014; Lee et al., 2015). Continued evolution of these viruses resulted in a third subclade (2.3.2.1c) in 2012 (Le and Nguyen, 2014; Lee et al., 2015). In recent years, viruses of subclades 2.3.2.1a and 2.3.2.1c have extended their geographic range and are now also found in the central and southern parts of Vietnam (Le and Nguyen, 2014; Nguyen et al., 2017), whereas viruses of subclade 2.3.2.1b have not been isolated recently (Le and Nguyen, 2014). Viruses of clade 1 have evolved into subclades 1.1.1 and 1.1.2, which have been isolated from the southern and central regions of Vietnam (Le and Nguyen, 2014; Lee et al., 2015; Nguyen et al., 2017). In addition to the dynamic evolution of the HA gene, reassortment among the other seven viral RNA (vRNA) segments of Vietnamese HPAI H5N1 poultry viruses has been reported (Zhao et al., 2012; Nguyen et al., 2017). Reassortment resulted in the emergence of clade 2.3.4.4 H5N6 viruses in Vietnam and other Southeast Asian countries (Wong et al., 2015; Chu et al., 2016; Lee et al., 2017; Nguyen et al., 2017). Thus, several (sub)lineages of HPAI H5 influenza viruses have been co-circulating in Vietnam, providing ample opportunity for further reassortment and the potential emergence of viruses that may transmit readily to humans.

Here, we analyzed more than 3,000 samples obtained from avian species in Vietnam in 2009–2013, resulting in the isolation of a number of HPAI H5N1 influenza viruses. Characterization of the HPAI H5N1 samples by phylogenetic analysis, Sanger sequencing, and deep-sequencing of 42 of the samples revealed multiple genotypes and potentially mammalian-adapting amino acid changes in some of the viral subpopulations.

## Materials and Methods

### Cells

Human embryonic kidney (293T) cells were purchased from the American Type Culture Collection (ATCC) and maintained in Dulbecco’s modified Eagle’s medium with 10% fetal bovine serum and antibiotics.

### Isolation of H5N1 Viruses

Oropharyngeal and/or cloacal swabs were collected from apparently healthy, sick, or dead poultry or wild birds and stored in transport medium (DMEM with 0.15% BSA, 100 IU/ml penicillin–streptomycin, 0.5 μg/ml amphotericin B, 100 μg/ml gentamicin, 20 μg/ml ciprofloxacin, and 0.02 M HEPES). In addition, organ samples were collected from dead or euthanized animals.

Nine-to-eleven-day-old specific pathogen-free eggs were inoculated with individual samples (for samples obtained from sick or dead animals), or with pooled samples (up to 10 samples obtained from healthy animals) and incubated at 35°C for 24–48 h. Allantoic fluid was collected and tested by means of HA assays. For all hemagglutination-positive samples, vRNA was extracted by using a QIAamp vRNA Minikit (Qiagen, Hilden, Germany). One-step reverse transcriptase (RT)-PCR was performed by using the Superscript III high-fidelity RT-PCR Kit (ThermoFisher, Waltham, MA, United States) and oligonucleotides to conserved regions of the matrix (M) gene (M-73F, 5′-GTCAGGCCCCCTCAAAGC; M-242R, 5′-CGTCTACGCTGCAGTCC). For all influenza virus-positive samples, portions of the viral HA and neuraminidase (NA) genes were amplified as described previously (Hoffmann et al., 2001) and Sanger-sequenced for subtype identification.

All samples were collected as part of the routine site health surveillance by the National Institute of Hygiene and Epidemiology, Vietnam. No ethical license was required.

The isolation of surveillance samples was carried out in an enhanced biosafety level 3 (BSL 3+) laboratory at the University of Wisconsin–Madison. Procedures using embryonated chicken eggs did not require approval in accordance with Public Health Service (PHS) Policy on Humane Care and Use of Laboratory Animals.

### Viral Genomic Sequence Analysis

The consensus sequences of all viral genes were determined by Sanger or deep-sequencing. vRNAs were extracted, reverse-transcribed, and amplified with oligonucleotide primers designed to amplify all eight vRNA segments (Hoffmann et al., 2001) for Sanger sequencing, or with a mixture of primers F1 (5′-GTTACGCGCCAGCAAAAGCAGG), F2 (5′-GTTACGCGCCAGCGAAAGCAGG), and R1 (5′-GTTACGCGCCAGTAGAAACAAGG) for deep-sequencing, using the Superscript III high-fidelity RT-PCR Kit (Invitrogen).

For deep-sequencing, amplicons were purified with 0.45× volume AMPure XP beads (Beckman Coulter) and 0.5–1 μg was sheared to an average fragment size of 150 bp on a Bioruptor Pico sonicator (Diagenode). Libraries for next-generation sequencing were prepared using the end repair, A-tailing, and adaptor ligation NEBNext DNA library prep modules for Illumina (New England Biolabs) according to the manufacturer’s protocol. Multiplexed and barcoded libraries were sequenced on the Illumina HiSeq 2500 platform in a single-end 100 nt run format. Single-end 100 nt reads were first filtered with cutadapt (Martin, 2011) to remove low-quality sequences and adapters. Next, we performed a *de novo* assembly using Cap3 (Huang and Madan, 1999) to generate a consensus sequence for each vRNA segment of each sample. These consensus sequences were further processed by using the ViVan pipeline (Isakov et al., 2015). We configured the ViVan pipeline to trim the reads by using EA-Tools/fastq-mcf (Aronesty, 2013), with 200,000 reads used for subsampling, minimum read lengths of 16 nucleotides, and minimum quality threshold (Phred) scores of 30. Next, we modified the ViVan pipeline to use Flexbar (Roehr et al., 2017), to trim 10 base pairs at both ends of all reads. The ViVan pipeline used BWA (Li and Durbin, 2010) to align the reads to the reference sequences; it detected sequence variants by its own statistical procedure. We only considered sequence variants with a minimum frequency of 1% and at least 1,000 reads at the position where the variant was found.

The consensus nucleotide sequences of the isolated H5N1 viruses were submitted to GenBank under the following accession numbers: KX513109–KX513409, KX644099–KX644131.

### Phylogenetic Analysis

**FIGURE 1:**
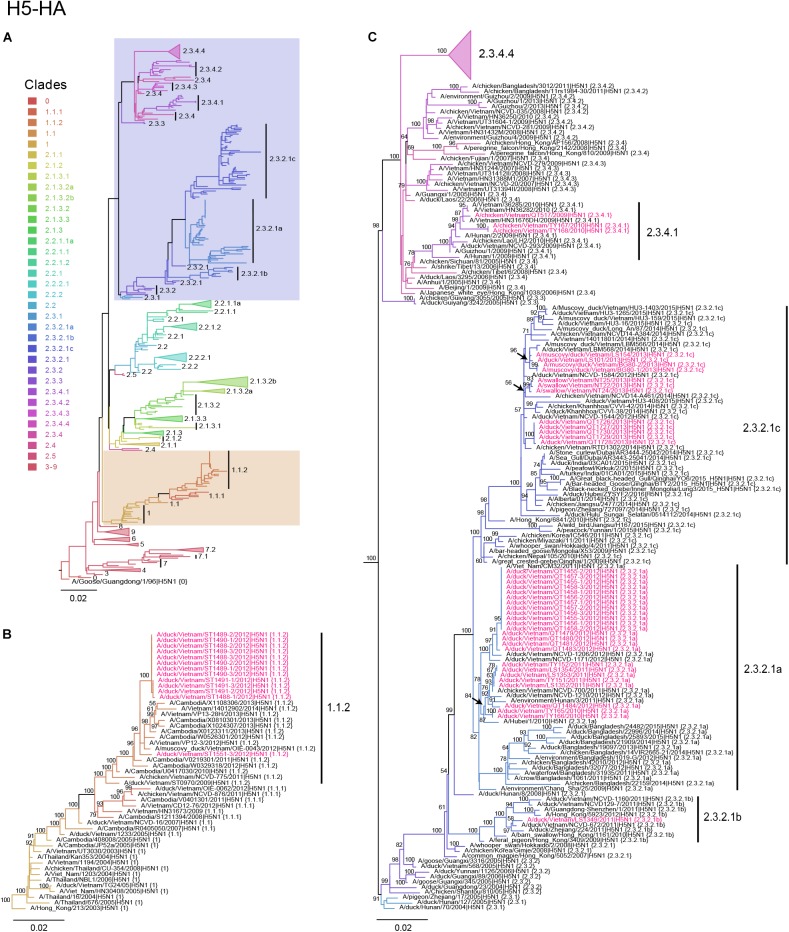
Phylogenetic analysis of the HA genes of HPAI H5N1 viruses. Evolutionary relationships of the H5 (HA) gene of influenza virus from 1996 to 2017, including avian and human viruses from different geographical regions. **(A)** H5-HA phylogeny reconstructed by using the ML method using 1,000 bootstrapping replicates. The tree was rooted using A/goose/Guangdong/1/96 (H5N1) virus. Branch color denotes different H5 clade designations. Shaded boxes indicate branches with avian H5N1 viruses isolated in this study. The scale bar represents nucleotide substitutions per site. **(B)** Magnification of H5 clade 1.1.2. Avian H5N1 sequences obtained in this study are shown in red. Bootstrap values greater than 50% are indicated at the nodes. The scale bar represents nucleotide substitutions per site. **(C)** Magnification of H5 clades 2.3.2.1a–2.3.2.1c and 2.3.4.1–2.3.4.3. Avian H5N1 sequences obtained in this study are shown in red. Bootstrap values above 50% are indicated at the nodes. The scale bar represents nucleotide substitutions per site.

Over 4,400 nucleotide sequences of H5Nx (for HA), HxN1 (for NA), and HxNx (for all internal gene segments) from 1996 to 2017 were downloaded from the NCBI Influenza Virus Resource and GISAID (accessed 23 August 2017; Supplementary Table S1). The datasets were aligned using MAFFT v.7.3 as implemented in Geneious Pro 9.0.3 (Biomatters Ltd.). The datasets were randomly sampled to produce smaller datasets and duplicate sequences were removed using custom scripts. In addition, new avian H5N1 virus sequences generated in this study were used for phylogenetic analysis, including 51 new sequences for the PB2 and PB1 vRNA segments and 53 new sequences for the PA, HA, NP, NA, M, and NS vRNA segments. The final datasets consisted of 346 sequences for the PB2 and PB1 vRNA segments; 425 for the H5-HA vRNA segment; 348 sequences for the PA, NP, M, and NS vRNA segments; and 221 sequences for the N1-NA vRNA RNA segment. To infer the evolutionary relationships of H5N1 viruses, individual gene phylogenies were reconstructed by using the maximum-likelihood (ML) method in RAxML v9.0 (Stamatakis, 2014), using a generalized time reversible nucleotide substitution model plus gamma distributed rates among sites (GTR+Γ), with 1,000 nonparametric bootstrap replicates for assessing branch support. The resulting H5 phylogeny (Figure 1) was subsequently annotated with H5 clade designations following the most recent update to the WHO/OIE/FAO H5 nomenclature system (Smith et al., 2015). The remaining gene topologies were shown in Supplementary Figures S1–S7.

### Minireplicon Assay

To determine the relative polymerase activities of A/duck/Vietnam/ST1488-3/2012 (H5N1) variants, consensus or variant genes of PB2, PB1, PA, and NP were inserted into the pCAGGS protein expression vector.

Human embryonic kidney (293T) cells were transfected with four protein expression plasmids for PB2, PB1, PA, and NP, with a plasmid for the expression of a virus-like RNA encoding the firefly luciferase gene under the control of the human RNA polymerase I promoter, and with a control plasmid encoding Renilla luciferase by using TransIT-LT1 (Mirus, Madison, WI, United States), and incubated for 48 h at 33 and 37°C, respectively. The cells were then lysed and the relative luciferase activity was measured by using a dual-luciferase reporter assay kit (Promega, Madison, WI, United States). Data shown are the mean values with standard deviations for the results of three independent experiments.

### Biosafety Statement

All recombinant DNA protocols were approved by the University of Wisconsin–Madison’s Institutional Biosafety Committee (IBC) after risk assessments were conducted by the Office of Biological Safety. All experiments were approved by the University of Wisconsin–Madison’s IBC. This manuscript was reviewed by the University of Wisconsin–Madison Dual Use Research of Concern (DURC) Subcommittee and by the Icahn School of Medicine at Mount Sinai (New York) IBC and DURC Committee. The reviews were conducted in accordance with the United States Government September 2014 DURC Policy. Both institutions concluded that the studies described herein do not meet the criteria of Dual Use Research (DUR) or DURC. In addition, the University of Wisconsin–Madison Biosecurity Task Force regularly reviews the research, policies, and practices of research conducted with pathogens of high consequence at the institution. This task force has a diverse skill set and provides support in the areas of biosafety, facilities, compliance, security, law, and health. Members of the Biosecurity Task Force are in frequent contact with the principal investigator and laboratory personnel to provide oversight and assure biosecurity.

**FIGURE 2:**
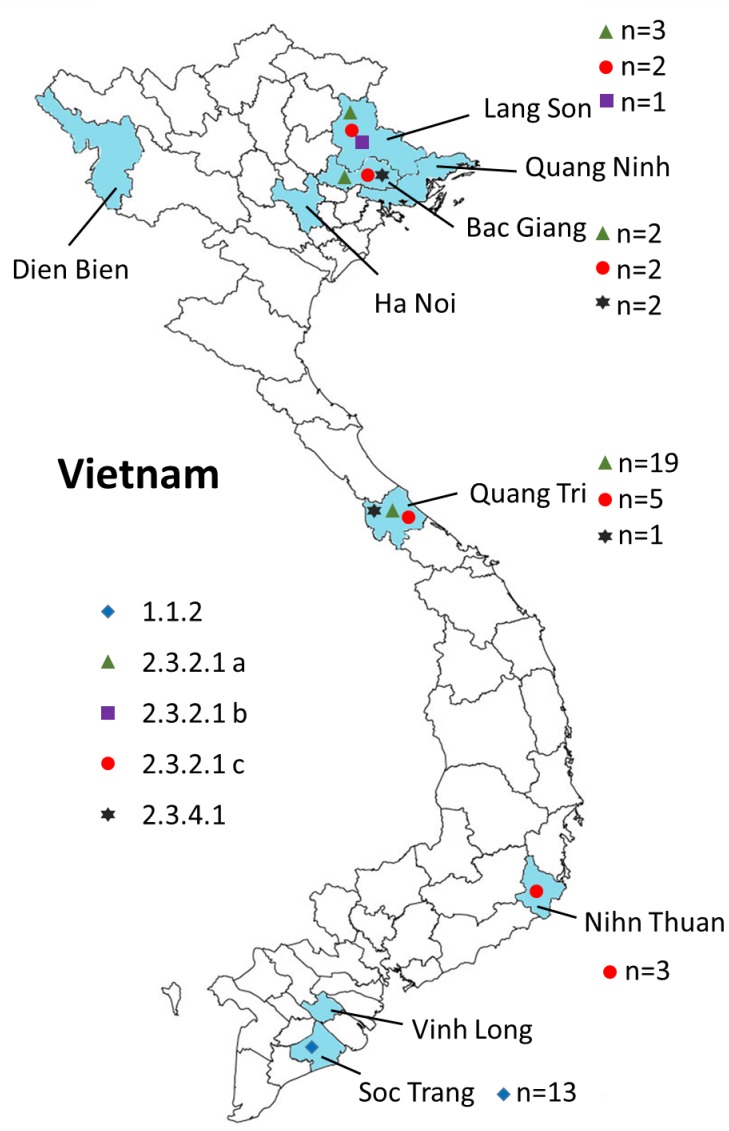
Geographical locations of the H5N1 viruses isolated from 2009 to 2013 in Vietnam. Provinces in which samples were collected are shown in blue. The virus clades are indicated by different symbols. Also shown are the numbers of isolates for each clade (*n* = *x*).

## Results and Discussion

### Isolation of Avian Influenza Viruses in Vietnam From 2009 to 2013

We carried out surveillance in live bird markets in different provinces of Vietnam (Figure 2) by collecting oropharyngeal swabs and cloacal samples from apparently healthy chickens and ducks (Table 1). In addition, after reports of suspected or confirmed outbreaks of HPAI H5N1 in backyard poultry flocks in rural villages, we obtained tissue samples from dead animals, and from sick animals that had to be euthanized. We also collected oropharyngeal and/or cloacal samples from apparently healthy poultry in the same village. Moreover, environmental samples and samples from dead wild birds (if available) were collected. In total, we collected 3,015 samples from 1,995 animals, including 95 tissue samples and 2,920 oropharyngeal and cloacal swabs (Table 1).

Samples obtained from sick or dead animals were amplified individually in 9–11-day-old embryonated chicken eggs, whereas we pooled up to 10 samples from healthy birds before inoculating them into embryonated chicken eggs. After amplification in embryonated chicken eggs, all samples were tested for hemagglutination activity. For hemagglutination-positive batches, individual samples were amplified in chicken eggs and then subjected to hemagglutination assays. Hemagglutination-positive samples were then tested for influenza viruses by PCR with oligonucleotides to a conserved region in the viral M segment. Next, portions of the HA and NA vRNAs were Sanger-sequenced to determine the influenza viral subtype. In total, we isolated 254 influenza viruses of five different subtypes: 53 H5N1 viruses, 34 H6N2 viruses, 34 H6N6 viruses, 3 H4N6 viruses, and 130 H9N2 viruses (Tables 1, 2). All H4, H6, and H9 viruses were isolated during surveillance from apparently healthy chickens and ducks in live bird markets (with the exception of one H9N2 virus, which was isolated from an apparently healthy duck during an outbreak response in a village). In contrast, 49 of the 53 H5N1 viruses were isolated from tissue samples from dead or euthanized animals from backyard flocks and wild birds in areas with suspected or reported HPAI H5N1 outbreaks. However, we also isolated one HPAI H5N1 virus from an apparently healthy chicken sampled in a village in the central province of Quang Tri, two HPAI H5N1 viruses from apparently healthy ducks sampled in a live bird market in the northern province of Lang Son, and one HPAI H5N1 virus from an apparently healthy swallow sampled in a village in the southern province of Ninh Thuan.

**Table 1:** Summary of surveillance samples and influenza viruses isolated.

Year	Surveillance type	Samples collected			Sample type		Geographical origin of samples			Influenza viruses isolated					Deep-sequencing^4^
		Animal	Health	Number of animals	Swab	Tissue	North^1^	Central^2^	South^3^	H5N1	H4N6	H6N2	H6N6	H9N2	
2009	Routine-LBM	Chickens	Healthy	71	141		141			0	0	0	0	0	
			Sick	8	16		16			0	0	0	0	0	
			Dead	14	27		27			0	0	0	0	0	
		Ducks	Healthy												
			Sick												
			Dead												
	Outbreak response (villages)	Chickens	Healthy	74	74		2	72		1	0	0	0	0	
			Sick												
			Dead												
		Ducks	Healthy	226	226		4	222		0	0	0	0	1	
			Sick												
			Dead												
2010	Routine-LBM	Chickens	Healthy	224	448		448			0	0	3	0	0	
			Sick												
			Dead												
		Ducks	Healthy	80	160		160			0	0	16	0	0	
			Sick												
			Dead												
		Geese	Healthy	5	10		10			0	0	0	0	0	
			Sick												
			Dead												
	Outbreak response (villages)	Chickens	Healthy												
			Sick												
			Dead	2		8		8		2	0	0	0	0	
		Ducks	Healthy												
			Sick												
			Dead	2		8		8		2	0	0	0	0	
		Geese	Healthy												
			Sick												
			Dead												
2011	Routine-LBM	Chickens	Healthy	189	378		378			0	0	0	4	0	
			Sick												
			Dead												
		Ducks	Healthy	285	570		170	400		0	3	0	0	0	
			Sick												
			Dead	2		2	2			2	0	0	0	0	
	Outbreak response (villages)	Chickens	Healthy												
			Sick												
			Dead												
		Ducks	Healthy												
			Sick												
			Dead	9		9	9			4	0	0	0	0	
2012	Routine-LBM	Chickens	Healthy	57	114		114			0	0	0	0	0	
			Sick												
			Dead												
		Ducks	Healthy	39	78			78		0	0	0	0	0	
			Sick												
			Dead												
	Outbreak response (villages)	Chickens	Healthy												
			Sick												
			Dead	5		15			15	0	0	0	0	0	
		Ducks	Healthy												
			Sick	8		8		8		5	0	0	0	0	5
			Dead	13		39		12	27	25	0	0	0	0	25
2013	Routine-LBM	Chickens	Healthy	360	360		331		29	0	0	6	5	99	
			Sick												
			Dead												
		Ducks	Healthy	311	311		264		47	2	0	9	25	30	2
			Sick												
			Dead												
	Outbreak response (villages)	Chickens	Healthy												
			Sick												
			Dead												
		Ducks	Healthy												
			Sick	6	2	5	15	5		7	0	0	0	0	7
			Dead												
		Swallow	Healthy	2	2	1^5^			3	1	0	0	0	0	1
			Sick												
			Dead	3	3				3	2	0	0	0	0	2
Total				1,995	2,920	95	1,346	813	124	53	3	34	34	130	42

*^1^Northern provinces of Lang Son, Dien Bien, Ha Noi, Quang Ninh, Bac Giang. ^2^Central province (Quang Tri). ^3^Southern provinces of Soc Trang, Vinh Long, Ninh Thuan. ^4^Viruses selected for deep-sequencing. ^5^Collected at a live market.*

**Table 2:** HPAI H5N1 viruses isolated from 2009 to 2013 in Vietnam.

Virus name	Sample type	Province	Region of Vietnam	Subclade	Deep-Sequenced	Total number of SNPs^1^
A/chicken/Vietnam/QT517/2009^2^	Swab^3^	Quang Tri	Central	2.3.4.1	No	N/A^4^
A/chicken/Vietnam/TY167/2010	Lung	Bac Giang	North	2.3.4.1	No	N/A
A/chicken/Vietnam/TY168/2010	Lung	Bac Giang	North	2.3.4.1	No	N/A
A/duck/Vietnam/TY165/2010	Lung	Quang Tri	Central	2.3.2.1a	No	N/A
A/duck/Vietnam/TY166/2010	Lung	Quang Tri	Central	2.3.2.1a	No	N/A
A/duck/Vietnam/LS1349/2011	Lung	Lang Son	North	2.3.2.1b	No	N/A
A/duck/Vietnam/LS1352/2011	Lung	Lang Son	North	2.3.2.1a	No	N/A
A/duck/Vietnam/LS1353/2011	Liver	Lang Son	North	2.3.2.1a	No	N/A
A/duck/Vietnam/LS1354/2011	Intestine	Lang Son	North	2.3.2.1a	No	N/A
A/duck/Vietnam/TY151/2011	Lung	Bac Giang	North	2.3.2.1a	No	N/A
A/duck/Vietnam/TY152/2011	Lung	Bac Giang	North	2.3.2.1a	No	N/A
A/duck/Vietnam/QT1455-1/2012	Liver	Quang Tri	Central	2.3.2.1a	Yes	4
A/duck/Vietnam/QT1455-2/2012	Lung	Quang Tri	Central	2.3.2.1a	Yes	4
A/duck/Vietnam/QT1455-3/2012	Heart	Quang Tri	Central	2.3.2.1a	Yes	4
A/duck/Vietnam/QT1456-1/2012	Liver	Quang Tri	Central	2.3.2.1a	Yes	5
A/duck/Vietnam/QT1456-2/2012	Intestine	Quang Tri	Central	2.3.2.1a	Yes	6
A/duck/Vietnam/QT1456-3/2012	Lung	Quang Tri	Central	2.3.2.1a	Yes	7
A/duck/Vietnam/QT1457-1/2012	Liver	Quang Tri	Central	2.3.2.1a	Yes	5
A/duck/Vietnam/QT1457-2/2012	Intestine	Quang Tri	Central	2.3.2.1a	Yes	26
A/duck/Vietnam/QT1457-3/2012	Lung	Quang Tri	Central	2.3.2.1a	Yes	9
A/duck/Vietnam/QT1458-1/2012	Liver	Quang Tri	Central	2.3.2.1a	Yes	8
A/duck/Vietnam/QT1458-2/2012	Intestine	Quang Tri	Central	2.3.2.1a	Yes	7
A/duck/Vietnam/QT1458-3/2012	Lung	Quang Tri	Central	2.3.2.1a	Yes	3
A/duck/Vietnam/QT1479/2012	Trachea	Quang Tri	Central	2.3.2.1a	Yes	10
A/duck/Vietnam/QT1480/2012	Trachea	Quang Tri	Central	2.3.2.1a	Yes	6
A/duck/Vietnam/QT1481/2012	Trachea	Quang Tri	Central	2.3.2.1a	Yes	3
A/duck/Vietnam/QT1483/2012	Trachea	Quang Tri	Central	2.3.2.1a	Yes	8
A/duck/Vietnam/QT1484/2012	Trachea	Quang Tri	Central	2.3.2.1a	Yes	12
A/duck/Vietnam/ST1488-1/2012	Trachea	Soc Trang	South	1.1.2	Yes	8
A/duck/Vietnam/ST1488-2/2012	Lung	Soc Trang	South	1.1.2	Yes	8
A/duck/Vietnam/ST1488-3/2012	Spleen	Soc Trang	South	1.1.2	Yes	11
A/duck/Vietnam/ST1489-1/2012	Trachea	Soc Trang	South	1.1.2	Yes	10
A/duck/Vietnam/ST1489-2/2012	Lung	Soc Trang	South	1.1.2	Yes	7
A/duck/Vietnam/ST1489-3/2012	Spleen	Soc Trang	South	1.1.2	Yes	8
A/duck/Vietnam/ST1490-1/2012	Trachea	Soc Trang	South	1.1.2	Yes	17
A/duck/Vietnam/ST1490-2/2012	Lung	Soc Trang	South	1.1.2	Yes	6
A/duck/Vietnam/ST1490-3/2012	Spleen	Soc Trang	South	1.1.2	Yes	3
A/duck/Vietnam/ST1491-1/2012	Trachea	Soc Trang	South	1.1.2	Yes	6
A/duck/Vietnam/ST1491-2/2012	Lung	Soc Trang	South	1.1.2	Yes	3
A/duck/Vietnam/ST1491-3/2012	Spleen	Soc Trang	South	1.1.2	Yes	5
A/duck/Vietnam/ST1551-3/2012	Liver	Soc Trang	South	1.1.2	Yes	4
A/swallow/Vietnam/NT22/2013	Swab^5^	Ninh Thuân	South	2.3.2.1c	Yes	11
A/swallow/Vietnam/NT24/2013	Swab^5^	Ninh Thuân	South	2.3.2.1c	Yes	15
A/swallow/Vietnam/NT25/2013^2^	Swab^6^	Ninh Thuân	South	2.3.2.1c	Yes	29
A/muscovy duck/Vietnam/BG80-1/2013	Swab^3^	Bac Giang	North	2.3.2.1c	Yes	12
A/muscovy duck/Vietnam/BG80-2/2013	Swab^6^	Bac Giang	North	2.3.2.1c	Yes	11
A/duck/Vietnam/QT1726/2013	Trachea	Quang Tri	Central	2.3.2.1c	Yes	17
A/duck/Vietnam/QT1727/2013	Trachea	Quang Tri	Central	2.3.2.1c	Yes	17
A/duck/Vietnam/QT1728/2013	Trachea	Quang Tri	Central	2.3.2.1c	Yes	3
A/duck/Vietnam/QT1729/2013	Trachea	Quang Tri	Central	2.3.2.1c	Yes	11
A/duck/Vietnam/QT1730/2013	Trachea	Quang Tri	Central	2.3.2.1c	Yes	10
A/duck/Vietnam/LS101/2013^2^	Swab^5^	Lang Son	North	2.3.2.1c	Yes	11
A/muscovy duck/Vietnam/ LS154/2013^2^	Swab^5^	Lang Son	North	2.3.2.1c	Yes	16

*^1^Total number of non-synonymous SNPs found at a frequency ≥1% that passed our quality control (see the section “Materials and Methods”). ^2^Sample was obtained from a healthy bird. ^3^Oropharyngeal swab. ^4^Not applicable. ^5^Sample is a mixture of cloacal and oropharyngeal swabs. ^6^Cloacal swab.*

### Phylogenetic Analysis of H5N1 Viruses

To understand the phylogenetic relationships of the novel HPAI H5N1 isolates, we analyzed complete genomes of the circulating H5N1 and H5Nx viruses of the Gs/GD (A/goose/Guangdong/1/1996 [Gs/GD] lineage in combination with 53 novel genomes generated from this study (Table 2). ML phylogenies were reconstructed for individual gene segments. The HA-H5 phylogeny (Figure 1A) consistently showed an extensive genetic diversification of the Gs/GD lineage worldwide. Our HA tree also indicated that the new H5 isolates from Vietnam were phylogenetically separated into different clades (Figure 1B,C), particularly in clades 1.1.2, 2.3.2.1a, 2.3.2.1b, 2.3.2.1c, and 2.3.4.1. Thirteen of the HPAI H5N1 viruses isolated here belonged to clade 1.1.2 and they were closely related to viruses from Vietnam and Cambodia. All new viruses in this clade were isolated in 2012 in the southern part of the country (Figure 1, 2 and Table 2), consistent with previous reports of the circulation of clade 1.1.2 viruses primarily in the southern part of Vietnam (Nguyen et al., 2012; Okamatsu et al., 2013). The clade 1.1.2 viruses characterized here were collected during the same outbreak, explaining the close phylogenetic relationship of most of these isolates.

Viruses of clade 2.3.4 and its subclades were frequently detected in the northern part of Vietnam until 2010 (Dung Nguyen et al., 2008; Nguyen et al., 2012; Le and Nguyen, 2014). We isolated three viruses of clade 2.3.4.1 from the northern and central provinces of Vietnam in 2009 and 2010 (Figure 1, 2 and Table 2). Viruses of clade 2.3.4 were then replaced by viruses of clade 2.3.2.1, which subsequently diverged into three subgroups (Creanga et al., 2013; Okamatsu et al., 2013; Le and Nguyen, 2014; Lee et al., 2015). Here, we isolated 24 H5N1 viruses of clade 2.3.2.1a from the central and northern part of the country in 2010–2012 (Figure 1, 2 and Table 2). In contrast, we isolated only a single virus of clade 2.3.2.1b (Figure 1, 2 and Table 2), which is consistent with a previous report that these viruses have not become dominant in Vietnam (Nguyen et al., 2017). In 2012, viruses of clade 2.3.2.1c emerged and have become dominant (Nguyen et al., 2017). This is consistent with our finding that all 12 HPAI H5N1 viruses isolated in 2013 belong to this subclade (Figure 1, 2 and Table 2); these viruses were isolated from northern, central, and southern provinces of Vietnam, demonstrating the wide geographic range of clade 2.3.2.1c viruses in Vietnam in 2013.

Next, we performed phylogenetic analyses for the remaining seven vRNA segments (Supplementary Figures S1–S7). The phylogenetic relationships of several vRNA segments (i.e., PB1, PA, NP, and NA) were found to be similar to those of the HA segment in that viruses of clades 1.1.2, 2.3.4.1, 2.3.2.1a, 2.3.2.1b, and 2.3.2.1c form separate groups. In contrast, we detected two separate phylogenetic groups for the PB2 segments of 2.3.2.1c viruses, the M segments of clades 2.3.2.1a and 2.3.2.1c viruses, and the NS segments of clade 2.3.2.1a viruses. These findings indicate reassortment among viruses of clade 2.3.2.1, but not between viruses of clades 1.1.2 and 2.3.2.1.

### Genetic Analysis of Vietnamese H5N1 Consensus Sequences

First, we assessed the consensus amino acid sequences of the viral proteins (Supplementary Table S2); our analysis focused on viruses of the major clades 1.1.2, 2.3.2.1a, and 2.3.2.1c (we did not perform a detailed analysis for the three clade 2.3.4.1 viruses and the single clade 2.3.21b virus because viruses of these clades have not been detected since 2010 and 2011, respectively). In general, many of the amino acid differences were detected at positions that separate the virus clades analyzed here (Supplementary Table S2). For many of these amino acid differences, we currently do not know their effect on virus replication, virulence, and/or antigenicity.

The HA protein possesses multiple basic amino acids at the cleavage site, a hallmark of HPAI viruses. Compared to the majority of H5 HA proteins, most clade 1.1.2 HA proteins encode a glutamic acid insertion immediately upstream of the HA cleavage site (based on our analysis of sequences available in the Influenza Research Database ^3^), which we also detected among the clade 1.1.2 viruses in the present study (Table 3 and Supplementary Table S2). All H5N1 HA proteins analyzed here encode glutamic acid and glycine at 222 and 224 (HA amino acid positions are based on H5 numbering; Burke and Smith, 2014), respectively, which confer preferential binding to α-2,3-linked sialic acids, that is, avian-type receptors (Wright et al., 2013). At positions 154–156, the clade 1.1.2 HA proteins encode the glycosylation motif “NST,” whereas the clade 2.3.2.1a,c HA proteins lack this glycosylation motif (they encode “DNA” at the respective amino acid positions) (Table 4). Loss of this glycosylation site may increase affinity for α-2,6-linked sialic acids (i.e., human-type receptors) and transmissibility among mammals via respiratory droplets (Herfst et al., 2012; Imai et al., 2012). In addition, viruses of clades 1.1.2, 2.3.2.1a, and 2.3.2.1c differ at HA positions that are part of the receptor-binding site (RBS; position 219), are located at the rim of the RBS (positions 129, 136, 140, 154–156, 184, and 189), affect HA receptor-binding specificity [positions 94 (Su et al., 2008), 120 (Wang et al., 2010), 123 (Yamada et al., 2006), and 210 (Watanabe et al., 2011)], or may affect HA stability [positions 162 (Kaverin et al., 2015) and 528 (Rudneva et al., 2013)]. Collectively, these differences may affect the virulence and host range of the respective viruses.

The NA protein encodes a sialidase whose activity is important for efficient virus release from host cells. In addition, NA contributes to the antigenic properties of viruses. The NA proteins characterized here possess a 20-amino acid deletion at positions 49–68 (Tables 3, 4 and Supplementary Table S2), which is typical for H5N1 NA proteins isolated since 2003 and increases H5N1 virulence in mice (Matsuoka et al., 2009; Zhou et al., 2009). The amino acid at position 309 is located in a pocket on the surface of NA, and an asparagine-to-serine mutation at this position was identified in an antigenic escape variant (Wan et al., 2013). Viruses of clade 1.1.2 encode NA-289(309)N (numbers indicate the amino acid position with and without the 20-amino acid deletion, respectively), whereas those of clades 2.3.2.1a,c encode NA-289(309)D (Tables 3, 4 and Supplementary Table S2); we currently do not know if the aspartic acid in this position affects the antigenic properties of NA. The emergence of NA variants with resistance to NA inhibitors is a major public health concern. Most clade 2.3.2.1a viruses characterized in the present study encode NA-106(126)N, whereas clades 1.1.2 and 2.3.2.1b viruses possess histidine at this position (Tables 3, 4 and Supplementary Table S2). The NA-H106(126)N mutation resulted in slightly increased (although not high) resistance to the NA inhibitors oseltamivir and zanamivir (Sheu et al., 2008), suggesting that clade 2.3.2.1a viruses may possess slightly reduced sensitivity to commonly used NA inhibitors.

**Table 3:** Comparison of selected amino acid residues of H5N1 viruses isolated in Vietnam.

Protein	Amino acid position	Phenotype, function, and/or location	Reference or other source	Clade 1.1.2		Clade 2.3.2.1a		Clade 2.3.2.1c		H5N1 consensus^1^
				This study	Consensus^1^	This study	Consensus^1^	This study	Consensus^1^	
PB2	147	Polymerase activity and virulence	Fan et al. (2014) (REF)	I	I	T	T	T	T	I
	271	Polymerase activity and virulence	Bussey et al. (2010) (REF)	M	T	T	T	T	T	T
	339	Polymerase activity and virulence	Fan et al. (2014) (REF)	K	K	T	T	T	T	K
	588	Polymerase activity and virulence	Fan et al. (2014) (REF)	A	A	T	A	T	T	A
	627	Polymerase activity and virulence	Subbarao et al. (1993) (REF)	E	E	E	E	E	E	E
			Hatta et al. (2001) (REF)							
	701	Polymerase activity and virulence	Li et al. (2005) (REF)	N	N	N	N	N	N	N
PB1-F2	66	Virulence in mice	Conenello et al. (2007) (REF)	S	S	N	N	N/truncated	N	N
PA	101	Polymerase activity and virulence	Hu et al. (2013) (REF)	E	E	D	D	N	N	E
	204	Polymerase activity	Liu et al. (2016) (REF)	K	K	R	R	R	R	K
	237	Polymerase activity and virulence	Hu et al. (2013) (REF)	K	K	E	E	K	E	E
	343	Polymerase activity and virulence	Zhong et al. (2018) (REF)	A	A	A/S	A	S	S	A
	391	Polymerase activity	Liu et al. (2016) (REF)	R	R	K	K	K	K	R
HA	94	Receptor-binding specificity	Su et al. (2008) (REF)	V	V	N	N	N	N	N
	120	Receptor-binding specificity	Wang et al. (2010) (REF)	S	S	D	D	D	D	S
	123	Receptor-binding specificity	Yamada et al. (2006) (REF)	P	P	T/S	S	S/P	S	S
	129	Rim of RBS	Structural analysis	M	L	L	L	L	L	L
	136	Rim of RBS	Structural analysis	P	P	P	P	S	S	P
	140	Rim of RBS	Structural analysis	Q	Q	N	N	N	N	N
	154	Rim of RBS; glycosylation site	Structural analysis	N	N	D	D	D	D	N
			Imai et al. (2012) (REF)							
	155	Rim of RBS; glycosylation site	Structural analysis	S	S	N	N	N	N	N
	156	Rim of RBS; glycosylation site	Structural analysis	T	T	A	A	A	A	T
			Herfst et al. (2012) (REF)							
	162	HA stability	Kaverin et al. (2015) (REF)	R	R	K	K	K	K	R
	184	Rim of RBS	Structural analysis	A	A	E	E	E	E	A
	189	Rim of RBS	Structural analysis	K	K	R/K	R	R	R	K
	210	Receptor-binding specificity	Watanabe et al. (2011) (REF)	T	T	V	V	V	V	V
	219	RBS	Structural analysis	V/I	V	I	I	I	I	V
	222	RBS	Structural analysis	E	E	E	E	E	E	E
	224	RBS	Structural analysis	G	G	G	G	G	G	G
	325–330	HA cleavage site		REERRKKR	REERRKKR	RE_RRRKR	RE_RRRKR	RE_RRRKR	RE_RRRKR	RERRRKKR
	528	HA stability, fusion activity, virulence	Rudneva et al. (2013) (REF)	A	A	V	V	V	V	A
NP	105	Pathogenicity in chickens	Tada et al. (2011) (REF)	M	V	V	V	V	V	V
NA^2^	Δ49–68	Enhanced virulence in mice	Matsuoka et al. (2009) (REF)	Deletion	Deletion	Deletion	Deletion	Deletion	Deletion	Deletion
			Zhou et al. (2009) (REF)							
	106 (126)	Sensitivity to neuraminidase inhibitors	Sheu et al. (2008) (REF)	H	H	N/H	H	H	H	H
	289 (309)	Antigenic escape variant	Wan et al. (2013) (REF)	N	N	D	N	D	N	N
M1	205	Viral growth properties	Yasuda et al. (1994) (REF)	I	I	V	I	V	V	V
M2	26	Resistance to ion channel inhibitors	Hay et al. (1986) (REF)	I	I	L	L	L	L	L
	27	Resistance to ion channel inhibitors	Hay et al. (1985) (REF)	V	V	V	V	I	V	V
	31	Resistance to ion channel inhibitors	Hay et al. (1985) (REF)	N	N	S	S	S	S	S
NS1^3^	Δ80–84	Enhanced virulence in mice	Long et al. (2008) (REF)	Deletion	Deletion	Deletion	Deletion; recent viruses lack deletion	Deletion	Deletion	Deletion
	87 (92)	Resistance to antiviral host responses	Seo et al. (2002) (REF)	D	D	E/D	D	E	E	D
	200 (205)	Resistance to antiviral host responses	Imai et al. (2010) (REF)	S	S	S	S	N	N	S
	222–225 (227–230)	Virulence in mice	Jackson et al. (2008) (REF)	ESEV	ESEV	ESEV	ESEV	ESEV	ESEV	ESEV

*^1^Based on sequences in the Influenza Virus Database (www.fludb.org). ^2^Numbers indicate amino acid position with (without) deletion of amino acids 49–68. ^3^Numbers indicate amino acid position with (without) deletion of amino acids 80–84.*

The PB2 protein (one of the viral polymerase subunits) is a major determinant of host range and pathogenicity (Subbarao et al., 1993; Hatta et al., 2001; Li et al., 2005; Shaw and Palese, 2013). The H5N1 viruses studied here encode PB2-627E (Subbarao et al., 1993; Hatta et al., 2001) and -701D (Li et al., 2005), that is, the amino acid residues typically found in avian influenza viruses (Table 3). However, the clade 2.3.2.1a,c viruses possess PB2-147T, -339T, and -588T (Tables 3, 4, and Supplementary Figure S1 and Supplementary Table S2), which we recently identified as novel determinants of avian H5N1 virulence in mammals (Fan et al., 2014). In contrast, viruses of clade 1.1.2 encode the avian-like sequences PB2-147I, -339K, and -588A. A PB2-A271T mutation increased the polymerase activity of an avian influenza virus polymerase in mammalian cells (Bussey et al., 2010). In addition, several computational studies identified the amino acid at position 271 of PB2 as host-specific, with avian influenza viruses typically encoding threonine, and human influenza viruses typically encoding alanine (Shaw et al., 2002; Chen et al., 2006; Finkelstein et al., 2007; Miotto et al., 2010). All clade 1.1.2 viruses characterized in the present study encode PB2-271M (Tables 3, 4, and Supplementary Figure S1 and Supplementary Table S2); this residue is found in 32 of the 89 clade 1.1.2 PB2 proteins deposited in the Influenza Research Database ^4^, with the remaining clade 1.1.2 PB2 proteins encoding threonine at this position (with the exception of one virus encoding alanine). We currently do not know the significance of the methionine residue found among clade 1.1.2 viruses at this position.

The PA protein encodes another subunit of the polymerase complex. The clade 1.1.2 and most of the clade 2.3.2.1a viruses analyzed here encode PA-343A (the most commonly found amino acid at this position) (Table 3 and Supplementary Table S2); in contrast, the clade 2.3.2.1c and some of the clade 2.3.2.1a viruses analyzed in our study encode PA-343S, which is prevalent among human influenza viruses of the H3N2 subtype. A PA-A343T substitution increased the replicative ability of an H5N1 virus in mammalian cells (Yamaji et al., 2015). Importantly, we recently characterized two of the viruses analyzed here (A/duck/Vietnam/QT1480/2012 and A/duck/Vietnam/QT1728/2013) and found that the PA-A343S mutation alone and in combination with a PA-D347E mutation significantly increased viral polymerase activity and mouse virulence (Zhong et al., 2018). In addition, viruses of clades 1.1.2, 2.3.2.1a, and 2.3.2.1c differ at several other amino acid positions in PA that affect the polymerase activity, including 204 (Liu et al., 2016), 237 (Hu et al., 2013), and 391 (Liu et al., 2016) (Table 3 and Supplementary Table S2).

The viral nucleoprotein NP of clade 1.1.2 viruses encodes a methionine at position 105, whereas most of the clade 2.3.21a,c viruses analyzed here possess NP-105V (Tables 3, 4 and Supplementary Table S2). The NP-M105V mutation increased the pathogenicity of an H5N1 virus in chickens (Tada et al., 2011), suggesting that the amino acid at this position contributes to H5N1 virulence in chickens.

In the viral M1 matrix protein, clade 1.1.2 viruses encode isoleucine at position 205 (Tables 3, 4 and Supplementary Table S2), which conferred high-growth properties to a reassortant human virus (Yasuda et al., 1994); in contrast, clades 2.3.2.1a and 2.3.2.1c viruses encode a valine residue, which is commonly found at this position among H5 influenza viruses. The viruses analyzed here also differ at several amino acid positions in the M2 ion channel protein that affect sensitivity to ion channel inhibitors (Hay et al., 1985, 1986): The M2-31N residue of clade 1.1.2 viruses (Tables 3, 4 and Supplementary Table S2) confers resistance to ion channel inhibitors; viruses encoding M2-31S (such as the clade 2.3.2.1a,c viruses) are sensitive to ion channel inhibitors. The amino acids found here at positions 26 and 27 of M2 (M2-26I/L or M2-27V/I, respectively) (Table 3 and Supplementary Table S2) also affect sensitivity to ion channel inhibitors (Lee et al., 2008; Chen and Guan, 2015).

**Table 4:** Selected mammalian-adapting and drug-resistance mutations in the HPAI H5N1 viruses isolated from 2009 to 2013 in Vietnam (shown are virulence markers that differ among the viruses characterized in this study or virulence markers associated with amino acid deletions).

Virus name	Subclade	Selected mammalian-adapting mutations^1^															Drug-resistance mutations	
		HA	NA	PB2		PA				NP	M1	NS1				PB1-F2	NA	M2
											
		154–156 Loss of glyc. site^2^	49–68 Δ^3^	147/339/588 T/T/T^4^	271 T^5^	204 K^6^	237 E^7^	343 S^8^	391 R^9^	105 V^10^	205 I^11^	80–84 Δ^12^	87 E^13^	200 S^14^	222–225 ESEV^15^	66 S^16^	106 N^17^	31 N^18^
A/chicken/Vietnam/QT517/2009	2.3.4.1		√		√		√		√	√		√		√	√			
A/chicken/Vietnam/TY167/2010			√		√		√		√	√		√		√	√			
A/chicken/Vietnam/TY168/2010			√		√		√		√	√		√		√	√			

A/duck/Vietnam/TY165/2010	2.3.2.1a	√	√	√	√		√			√		√		√	√			
A/duck/Vietnam/TY166/2010		√	√	√	√		√			√		√		√	√			

A/duck/Vietnam/LS1349/2011	2.3.2.1b	√	√	√	√		√			√		√	√	√	√			

A/duck/Vietnam/LS1352/2011	2.3.2.1a	√	√	√	√		√	√		√	√	√		√	√			
A/duck/Vietnam/LS1353/2011		√	√	√	√		√	√		√	√	√		√	√			
A/duck/Vietnam/ LS1354/2011		√	√	√	√		√	√		√	√	√		√	√			
A/duck/Vietnam/TY151/2011		√	√	√	√		√	√		√	√	√		√	√			
A/duck/Vietnam/TY152/2011		√	√	√	√		√	√		√	√	√		√	√			
A/duck/Vietnam/QT1455-1/2012		√	√	√	√		√			√		√	√	√	√		√	
A/duck/Vietnam/QT1455-2/2012		√	√	√	√		√			√		√	√	√	√		√	
A/duck/Vietnam/QT1455-3/2012		√	√	√	√		√			√		√	√	√	√		√	
A/duck/Vietnam/QT1456-1/2012		√	√	√	√		√			√		√	√	√	√		√	
A/duck/Vietnam/QT1456-2/2012		√	√	√	√		√			√		√	√	√	√		√	
A/duck/Vietnam/QT1456-3/2012		√	√	√	√		√			√		√	√	√	√		√	
A/duck/Vietnam/QT1457-1/2012		√	√	√	√		√			√		√	√	√	√		√	
A/duck/Vietnam/QT1457-2/2012		√	√	√	√		√			√		√	√	√	√		√	
A/duck/Vietnam/QT1457-3/2012		√	√	√	√		√			√		√	√	√	√		√	
A/duck/Vietnam/QT1458-1/2012		√	√	√	√		√			√		√	√	√	√		√	
A/duck/Vietnam/QT1458-2/2012		√	√	√	√		√			√		√	√	√	√		√	
A/duck/Vietnam/QT1458-3/2012		√	√	√	√		√			√		√	√	√	√		√	
A/duck/Vietnam/QT1479/2012		√	√	√	√		√			√		√	√	√	√		√	
A/duck/Vietnam/QT1480/2012		√	√	√	√		√					√	√	√	√		√	
A/duck/Vietnam/QT1481/2012		√	√	√	√		√					√	√	√	√		√	
A/duck/Vietnam/QT1483/2012		√	√	√	√		√			√		√	√	√	√		√	
A/duck/Vietnam/QT1484/2012		√	√	√	√		√	√		√		√		√	√			
A/duck/Vietnam/ST1488-1/2012			√			√			√		√	√		√	√	√		√
A/duck/Vietnam/ST1488-2/2012			√			√			√		√	√		√	√	√		√
A/duck/Vietnam/ST1488-3/2012			√			√			√		√	√		√	√	√		√
A/duck/Vietnam/ST1489-1/2012			√			√			√		√	√		√	√	√		√
A/duck/Vietnam/ST1489-2/2012			√			√			√		√	√		√	√	√		√
A/duck/Vietnam/ST1489-3/2012			√			√			√		√	√		√	√	√		√
A/duck/Vietnam/ST1490-1/2012	1.1.2		√			√			√		√	√		√	√	√		√
A/duck/Vietnam/ST1490-2/2012			√			√			√		√	√		√	√	√		√
A/duck/Vietnam/ST1490-3/2012			√			√			√		√	√		√	√	√		√
A/duck/Vietnam/ST1491-1/2012			√			√			√		√	√		√	√	√		√
A/duck/Vietnam/ST1491-2/2012			√			√			√		√	√		√	√	√		√
A/duck/Vietnam/ST1491-3/2012			√			√			√		√	√		√	√	√		√
A/duck/Vietnam/ST1551-3/2012			√			√			√	√	√	√		√	√	√		√

A/swallow/Vietnam/NT22/2013	2.3.2.1c	√	√	√	√			√		√		√	√		√			
A/swallow/Vietnam/NT24/2013		√	√	√	√			√		√		√	√		√			
A/swallow/Vietnam/NT25/2013		√	√	√	√			√		√		√	√		√			
A/muscovy duck/Vietnam/BG80-1/2013		√	√	√	√			√		√		√	√		√			
A/muscovy duck/Vietnam/BG80-2/2013		√	√	√	√			√		√		√	√		√			
A/duck/Vietnam/QT1726/2013		√	√	√	√			√		√		√	√		√			
A/duck/Vietnam/QT1727/2013		√	√	√	√			√		√		√	√		√			
A/duck/Vietnam/QT1728/2013		√	√	√	√			√		√		√	√		√			
A/duck/Vietnam/QT1729/2013		√	√	√	√			√		√		√	√		√			
A/duck/Vietnam/QT1730/2013		√	√	√	√			√		√		√	√		√			
A/duck/Vietnam/LS101/2013		√	√	√	√			√		√		√	√		√			
A/muscovy duck/Vietnam/ LS154/2013		√	√	√	√			√		√		√	√		√			

*^1^Empty cells: The particular mammalian-adapting mutation is not encoded by the respective virus isolate; checkmark: The particular mammalian-adapting mutation is encoded by the respective virus isolate. ^2^Indicates the loss of a glycosylation site (NxS/T) at positions 154–156 of HA; Imai et al. (2012) (24); Herfst et al. (2012) (25). ^3^Matsuoka et al. (2009) (32); Zhou et al. (2009) (33). ^4^Fan et al. (2014) (40). ^5^Bussey et al. (2010) (41). ^6^Liu et al. (2016) (48). ^7^Hu et al. (2013) (49). ^8^Zhong et al. (2018) (47). ^9^Liu et al. (2016) (48). ^10^Tada et al. (2011) (50). ^11^Yasuda et al. (1994) (51). ^12^Long et al. (2008) (56). ^13^Seo et al. (2002) (57). ^14^Imai et al. (2010) (58). ^15^Jackson et al. (2008) (59). ^16^Conenello et al. (2007) (63). ^17^Sheu et al. (2008) (35). ^18^Hay et al. (1985) (52).*

The viral NS1 protein counteracts cellular anti-viral interferon (IFN) responses (Wright et al., 2013). The NS1 proteins of all viruses characterized here lack amino acids 80–84 (Tables 3, 4 and Supplementary Table S2), a deletion that is commonly detected among influenza H5N1 NS1 proteins and confers increased H5N1 virulence in mice (Long et al., 2008). However, most clade 2.3.2.1a viruses collected since 2015 do not possess this deletion, indicating a reassortment event involving NS. At position 87 (equivalent to position 92 of NS1 proteins lacking the deletion), viruses of clade 1.1.2 encode NS1-87D, whereas most clade 2.3.2.1a,c viruses encode NS1-87E (Tables 3, 4 and Supplementary Table S2). The NS1-87(92)E residue confers enhanced resistance to the antiviral effects of IFN compared to NS1-87(92)D (Seo et al., 2002). At position 200(205), the clades 1.1.2 and 2.3.2.1a viruses possess a serine residue (Tables 3, 4 and Supplementary Table S2), which affects the antiviral host responses compared to NS1-200(205)N (Imai et al., 2010), the residue encoded by clade 2.3.2.1c viruses. All viruses characterized here possess a PDZ domain-binding motif with the sequence ESEV [positions 222–225 (227–230)] (Tables 3, 4 and Supplementary Table S2), which conferred high virulence to an influenza virus in mice (Jackson et al., 2008).

The PB1-F2 protein is a short viral protein encoded by the +1 alternate open-reading frame relative to the PB1 open-reading frame (Chen et al., 2001). It affects virulence by modulating host apoptosis and inflammation, and by interacting with PB1 (Conenello and Palese, 2007; Krumbholz et al., 2011). In particular, the PB1-F2-N66S mutation increases the virulence of influenza viruses (Conenello et al., 2007). Among the viruses characterized here, viruses of clade 1.1.2 encode PB1-F2-66S, whereas those of clade 2.3.2.1a encode PB1-F2-66N (Tables 3, 4 and Supplementary Table S1). Viruses of clade 2.3.2.1c fall into two groups: those encoding PB1-F2-66N or those encoding a premature stop codon that results in a truncated PB1-F2 protein of 57 amino acids (Table 3 and Supplementary Table S2).

### Analysis of Viral Subpopulations

Next, we assessed the viral subpopulations of the 42 H5N1 influenza viruses that were deep-sequenced (Table 2). We only considered sequence variants with a Phred quality score >30, a minimum frequency of 1%, and at least 1,000 reads at positions where variants were found. For our analysis, we focused on non-synonymous single-nucleotide polymorphisms (SNPs), that is, sequence variants that resulted in amino acid changes.

Applying the parameters described above, we detected the largest numbers of non-synonymous SNPs in the PB2, PB1, PA, and HA proteins (Supplementary Table S3). Given that M2 is only 97 amino acids, the number of non-synonymous SNPs in M2 was relatively high; however, almost all of these SNPs were detected in the three clade 2.3.2.1c viruses isolated from swallows. Currently, we do not know the significance of this finding. The fewest numbers of non-synonymous SNPs were detected in NS2 (a short viral protein of 121 amino acids that does not play a major role in host adaptation) and M1, which is relatively conserved among influenza A viruses.

Among the samples analyzed here, we detected a relatively high number of non-synonymous SNPs in a virus isolated from an apparently healthy swallow: 29 non-synonymous SNPs in A/swallow/Vietnam/NT25/2013 (Table 2). This large number of SNPs may reflect adaptation to a new host. Interestingly, barn swallows have been discussed as “bridge hosts” that may transfer influenza viruses between waterfowl and domestic avian species (Caron et al., 2014, 2017).

Next, we assessed the viral subpopulations for variants that may affect virulence (Supplementary Table S3). For example, we detected viral subpopulations with amino acid changes at HA positions located at the rim of the RBS (which may affect receptor-binding and antigenicity), or at the RBS. Similarly, for NA, we found viral subpopulations with changes at positions 96 (116) or 253 (273), which may affect sensitivity to oseltamivir and zanamivir (Hurt et al., 2007; Boltz et al., 2010), or antigenicity (Wan et al., 2013), respectively. Several samples also contained subpopulations of viruses encoding M2-S31N, which confers resistance to ion channel inhibitors (Supplementary Table S3). Other examples include viral subpopulations possessing NP-A105V, which increases H5N1 virulence in chickens (Tada et al., 2011), or M1-V205I, an amino acid change that confers high-growth properties (Yasuda et al., 1994) (Supplementary Table S3). We also compared the viral subpopulations of the avian samples with those of human H5N1 virus samples (Imai et al., 2018). Only one amino acid position (PA-142) showed sequence variability in both sets of samples; an amino acid change at this position significantly altered the polymerase activity (Imai et al., 2018).

**FIGURE 3:**
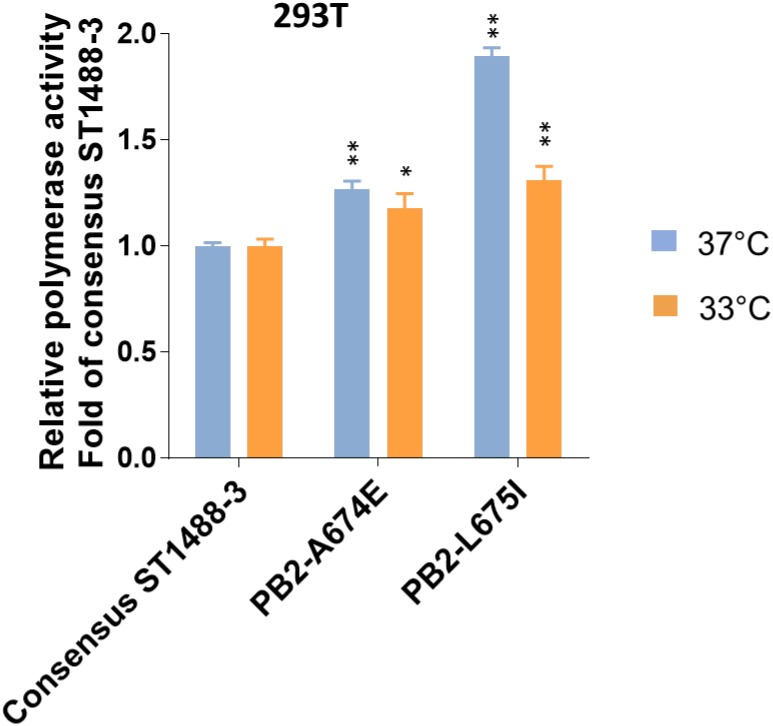
Effect of selected mutations detected by deep-sequencing on the viral polymerase activity. 293T cells were transfected with four plasmids expressing the PB1, PA, and NP proteins; consensus or variant PB2 protein of A/duck/Vietnam/ST1488-3/2012 (ST1488-3); a plasmid for the expression of a virus-like RNA encoding the firefly luciferase gene; and a control plasmid encoding Renilla luciferase, and assayed after a 48 h incubation at 33 and 37°C. Relative polymerase activity was determined and normalized to that of consensus ST1488-3 polymerase complexes. Data shown are mean values with standard deviations for the results of three independent experiments. *P*-values were calculated by one-way ANOVA, followed by Dunnett’s test (^∗^*P* < 0.05; ^∗∗^*P* < 0.01 compared to consensus ST1488-3).

### Functional Analysis of Mutations in Viral Subpopulations

To assess the functional effect of SNPs detected by deep-sequencing, we selected two mutations, A674E and L675I (Supplementary Table S3) in the PB2 protein of A/duck/Vietnam/ST1488-3/2012 (ST1488-3). These mutations were chosen because of their close proximity, their location on the surface of the protein, and the fact that the amino acid at position 674 may be host specific, based on computational analyses (Shaw et al., 2002; Chen et al., 2006; Finkelstein et al., 2007; Miotto et al., 2010). To assess a potential role of these sequence variants, we tested the polymerase activities of viral polymerase complexes in minireplicon assay in mammalian cells at 37 and 33°C. Introduction of mutation A674E or L675I into the consensus PB2 protein of ST1488-3 resulted in a small, but significant increase of polymerase activity in 293T cells at both temperatures (Figure 3). These results suggested that the sequence variants detected among H5N1 influenza viruses may play a biological role.

In summary, avian surveillance in Vietnam in 2009–2013 resulted in the isolation of a number of H5N1 viruses from different subclades. By 2013, viruses of clade 2.3.2.1c had become dominant and were isolated from different provinces in Vietnam. Deep-sequencing identified viral subpopulations with amino acid changes that may facilitate adaptation to mammals. A virus isolated from a healthy barn swallow encoded a high number of non-synonymous SNPs, suggesting that these viruses were under adaptive pressure in barn swallows.

## Author Contributions

ML, HN, and PVMH collected the surveillance samples. GZ, SF, PH, MH, and GN isolated the viruses, carried out Sanger sequencing, and/or analyzed the Sanger and deep-sequencing data. HvB and JD carried out deep-sequencing. TL processed the deep-sequencing data. YS, JJ, and GS performed the phylogenetic analyses. GZ, SF, GN, and YK planned the study and wrote the manuscript.

## Conflict of Interest Statement

YK and GN are co-founders of FluGen. The remaining authors declare that the research was conducted in the absence of any commercial or financial relationships that could be construed as a potential conflict of interest.

## Footnotes

1 http://www.oie.int/fileadmin/Home/eng/Animal_Health_in_the_World/docs/pdf/graph_avian_influenza/graphs_HPAI_01_11_2016.pdf; accessed on 14 December 2017

2 https://www.fludb.org

3 www.fludb.org; accessed on 10/06/2018

4 www.fludb.org; accessed on 24 November 2017

## Supplementary Material

FIGURE S1 Maximum-likelihood (ML) phylogeny of avian PB2 genes. Red font denotes H5N1 influenza viruses collected in Vietnam in this study. Bootstrap values greater than 50% are indicated at the nodes. The scale bar represents nucleotide substitutions per site. Vertical bars indicate (sub)clades and groups of viruses possessing mammalian-adapting amino acid changes.

FIGURE S2 ML phylogeny of avian PB1 genes. Red font denotes H5N1 influenza viruses collected in Vietnam in this study. Bootstrap values greater than 50% are indicated at the nodes. The scale bar represents nucleotide substitutions per site. Vertical bars indicate (sub)clades.

FIGURE S3 ML phylogeny of avian PA genes. Red font denotes H5N1 influenza viruses collected in Vietnam in this study. Bootstrap values greater than 50% are indicated at the nodes. The scale bar represents nucleotide substitutions per site. Vertical bars indicate (sub)clades.

FIGURE S4 ML phylogeny of avian NP genes. Red font denotes H5N1 influenza viruses collected in Vietnam in this study. Bootstrap values greater than 50% are indicated at the nodes. The scale bar represents nucleotide substitutions per site. Vertical bars indicate (sub)clades.

FIGURE S5 ML phylogeny of avian N1-NA genes. Red font denotes H5N1 influenza viruses collected in Vietnam in this study. Bootstrap values greater than 50% are indicated at the nodes. The scale bar represents nucleotide substitutions per site. Vertical bars indicate (sub)clades.

FIGURE S6 ML phylogeny of avian M genes. Red font denotes H5N1 influenza viruses collected in Vietnam in this study. Bootstrap values greater than 50% are indicated at the nodes. The scale bar represents nucleotide substitutions per site. Vertical bars indicate (sub)clades.

FIGURE S7 ML phylogeny of avian NS genes. Red font denotes H5N1 influenza viruses collected in Vietnam in this study. Bootstrap values greater than 50% are indicated at the nodes. The scale bar represents nucleotide substitutions per site. Vertical bars indicate (sub)clades.

TABLE S1 GISAID acknowledgments for PB2, PB1, PA, H5-HA, NP, N1-NA, MP, and NS genes.

TABLE S2 Comparison of the consensus sequences of the isolated viruses (the sequence of A/duck/Vietnam/ST1488-1/2012 served as the reference sequence).

TABLE S3 Summary of deep-sequencing data: shown are non-synonymous SNPs found at a frequency ≥1% that passed our quality control (see the section “Materials and Methods”); data are sorted by viral protein and, for each protein, by the frequency of the SNP.
