# Supplementary figures and images for: Isolation of Highly Pathogenic H5N1 Influenza Viruses in 2009–2013 in Vietnam
Source: Front Microbiol. 2019 Jun 25;10:1411. doi: 10.3389/fmicb.2019.01411 (PMC6603144; doi:10.3389/fmicb.2019.01411)

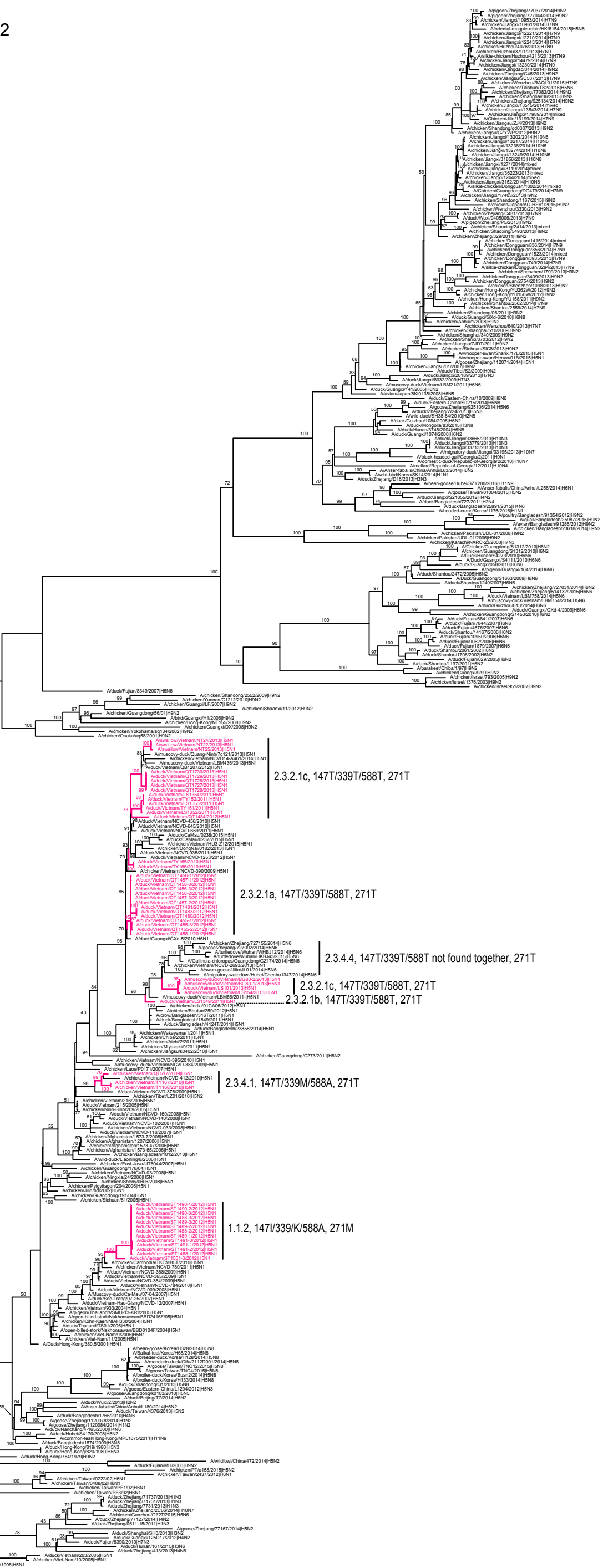

2.3.2.1c, 147T/339T/588T, 271T

2.3.2.1a, 147T/339T/588T, 271T

2.3.4.4, 147T/339T/588T not found together, 271T

2.3.2.1c, 147T/339T/588T, 271T

2.3.2.1b, 147T/339T/588T, 271T

2.3.4.1, 147T/339M/588A, 271T

1.1.2, 147I/339K/588A, 271M

2.3.2.1c, 147T/339T/588T, 271T

Supplement: FIGURE S1 — Maximum-likelihood (ML) phylogeny of avian PB2 genes. Red font denotes H5N1 influenza viruses collected in Vietnam in this study. Bootstrap values greater than 50% are indicated at the nodes. The scale bar represents nucleotide substitutions per site. Vertical bars indicate (sub)clades and groups of viruses possessing mammalian-adapting amino acid changes. [file Data_Sheet_1.PDF]

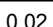

Supplement: FIGURE S2 — ML phylogeny of avian PB1 genes. Red font denotes H5N1 influenza viruses collected in Vietnam in this study. Bootstrap values greater than 50% are indicated at the nodes. The scale bar represents nucleotide substitutions per site. Vertical bars indicate (sub)clades. [file Data_Sheet_2.PDF]

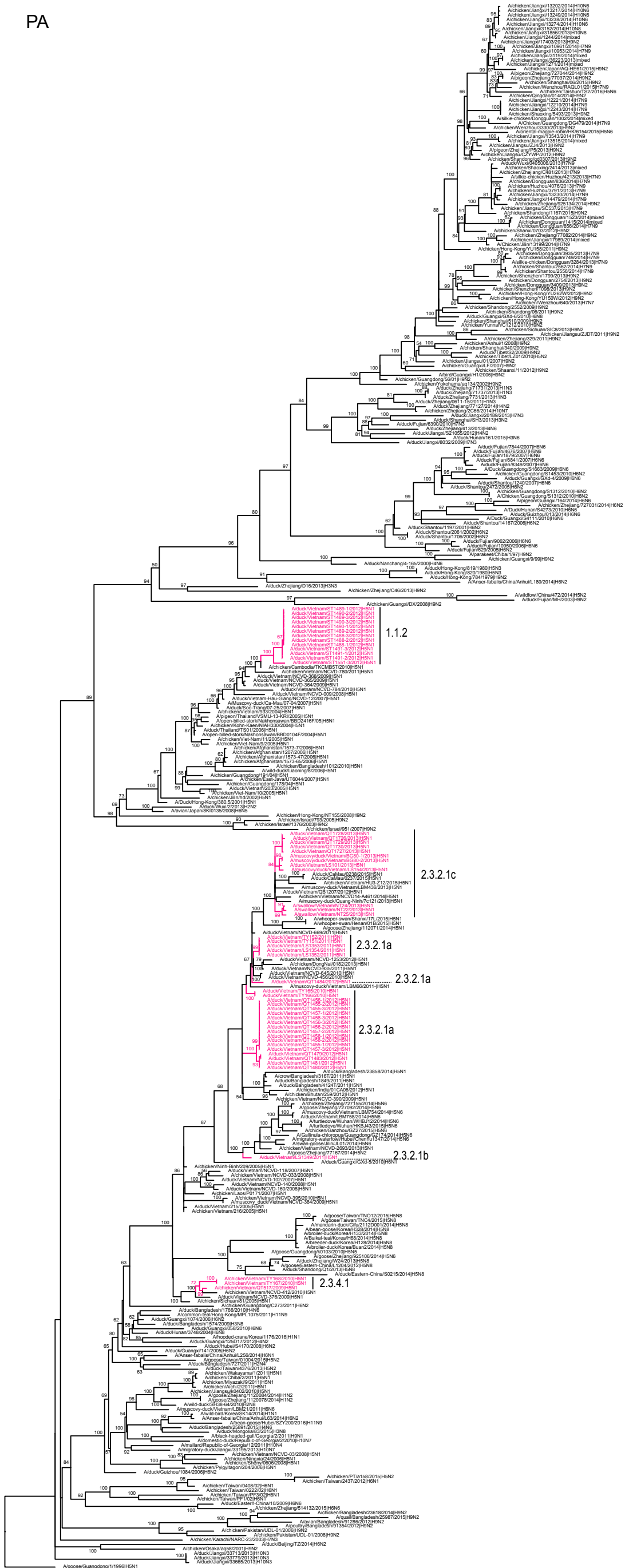

Supplement: FIGURE S3 — ML phylogeny of avian PA genes. Red font denotes H5N1 influenza viruses collected in Vietnam in this study. Bootstrap values greater than 50% are indicated at the nodes. The scale bar represents nucleotide substitutions per site. Vertical bars indicate (sub)clades. [file Data_Sheet_3.PDF]

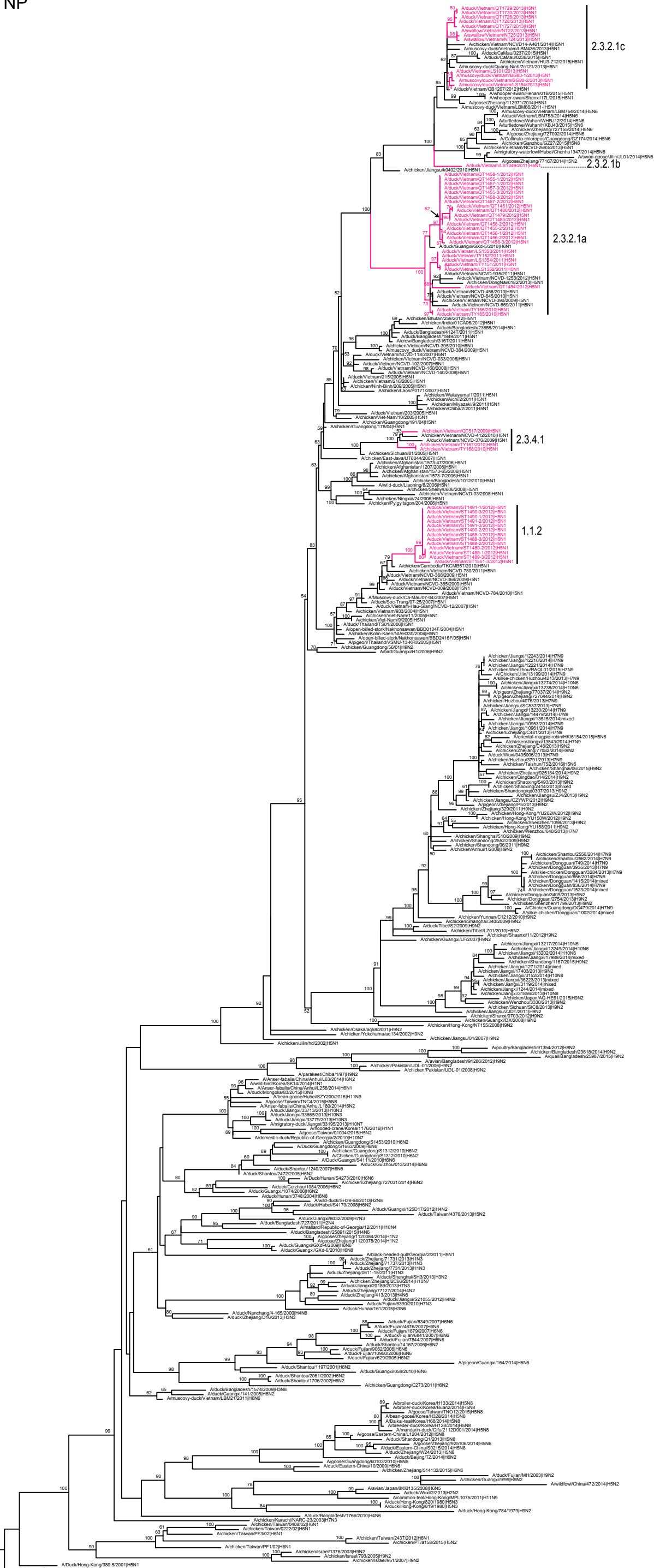

Supplement: FIGURE S4 — ML phylogeny of avian NP genes. Red font denotes H5N1 influenza viruses collected in Vietnam in this study. Bootstrap values greater than 50% are indicated at the nodes. The scale bar represents nucleotide substitutions per site. Vertical bars indicate (sub)clades. [file Data_Sheet_4.PDF]

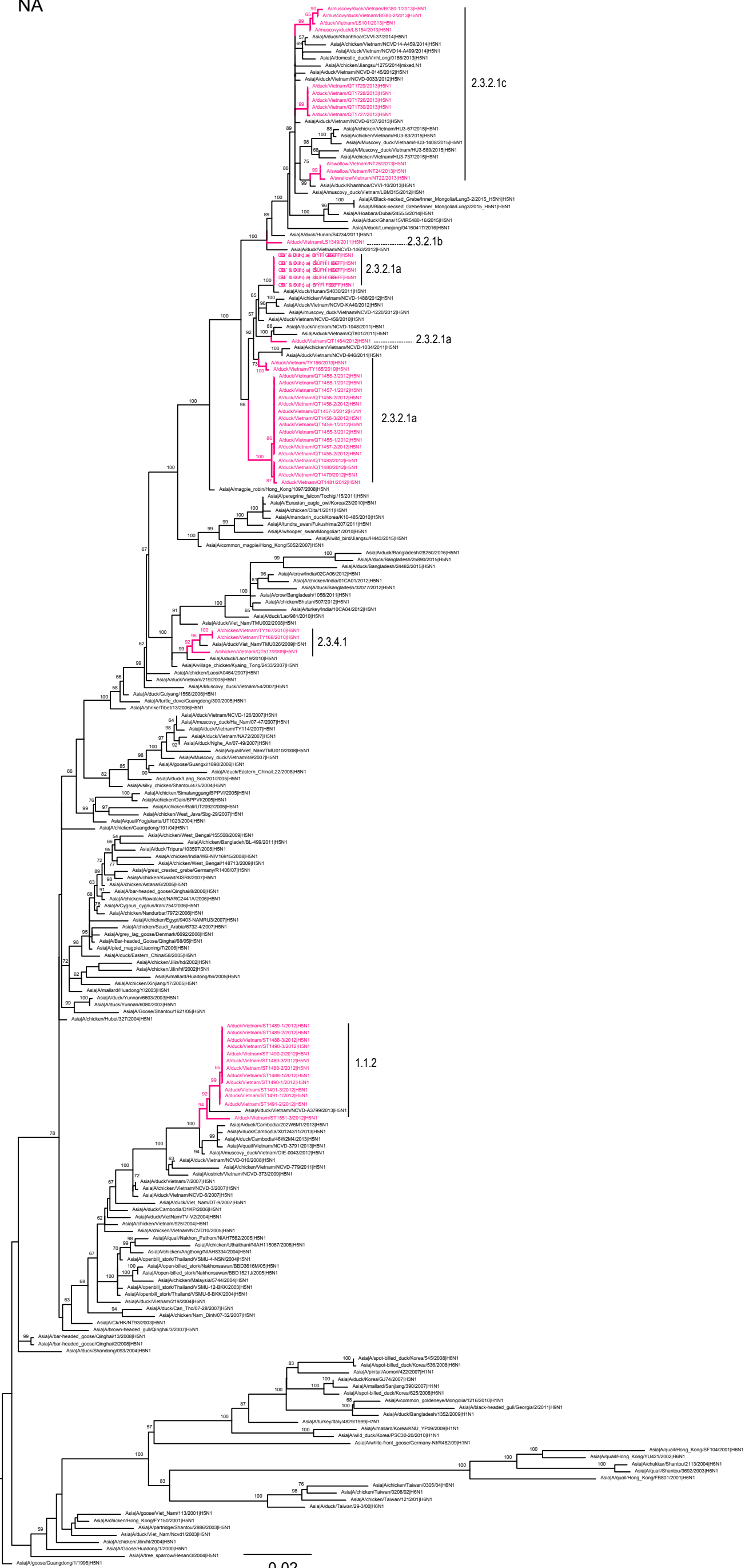

Supplement: FIGURE S5 — ML phylogeny of avian N1-NA genes. Red font denotes H5N1 influenza viruses collected in Vietnam in this study. Bootstrap values greater than 50% are indicated at the nodes. The scale bar represents nucleotide substitutions per site. Vertical bars indicate (sub)clades. [file Data_Sheet_5.PDF]

MP

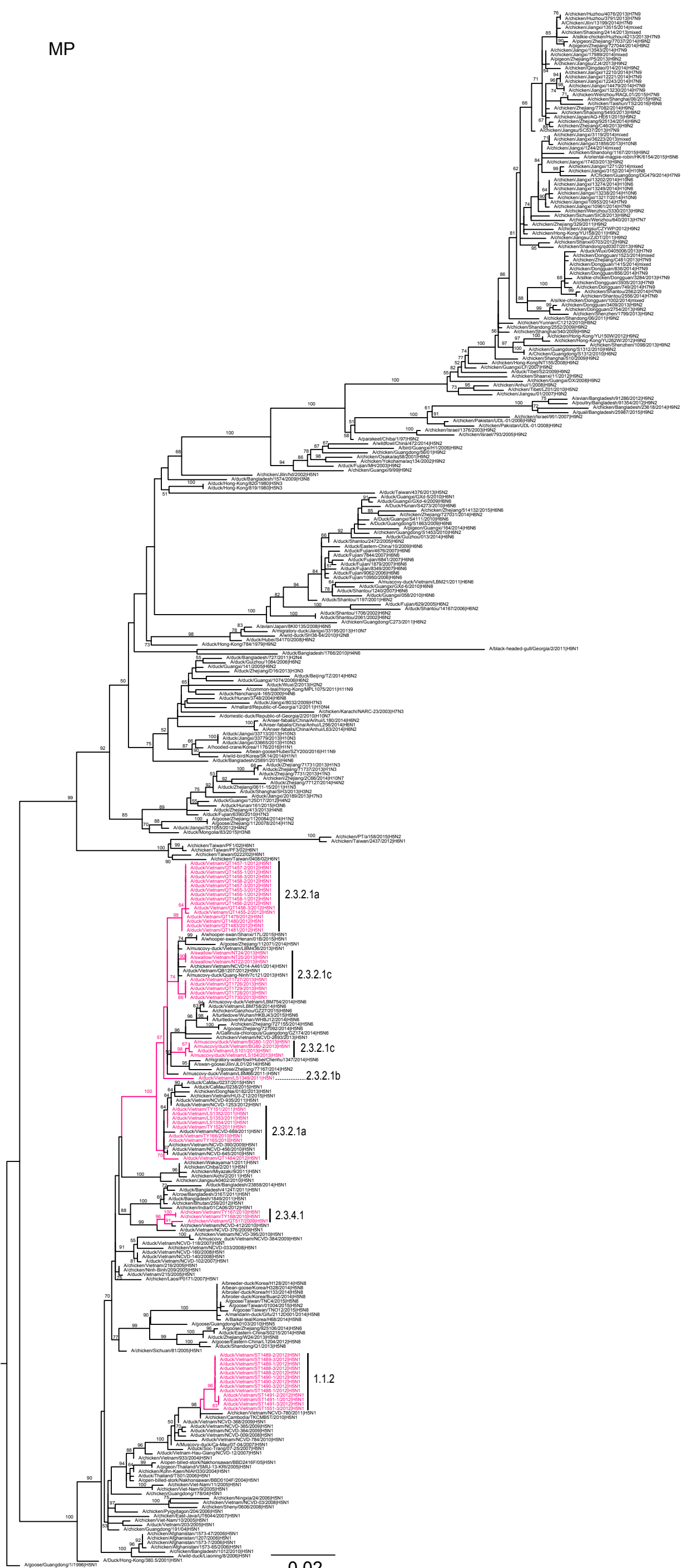

Supplement: FIGURE S6 — ML phylogeny of avian M genes. Red font denotes H5N1 influenza viruses collected in Vietnam in this study. Bootstrap values greater than 50% are indicated at the nodes. The scale bar represents nucleotide substitutions per site. Vertical bars indicate (sub)clades. [file Data_Sheet_6.PDF]

Allele B

Allele A

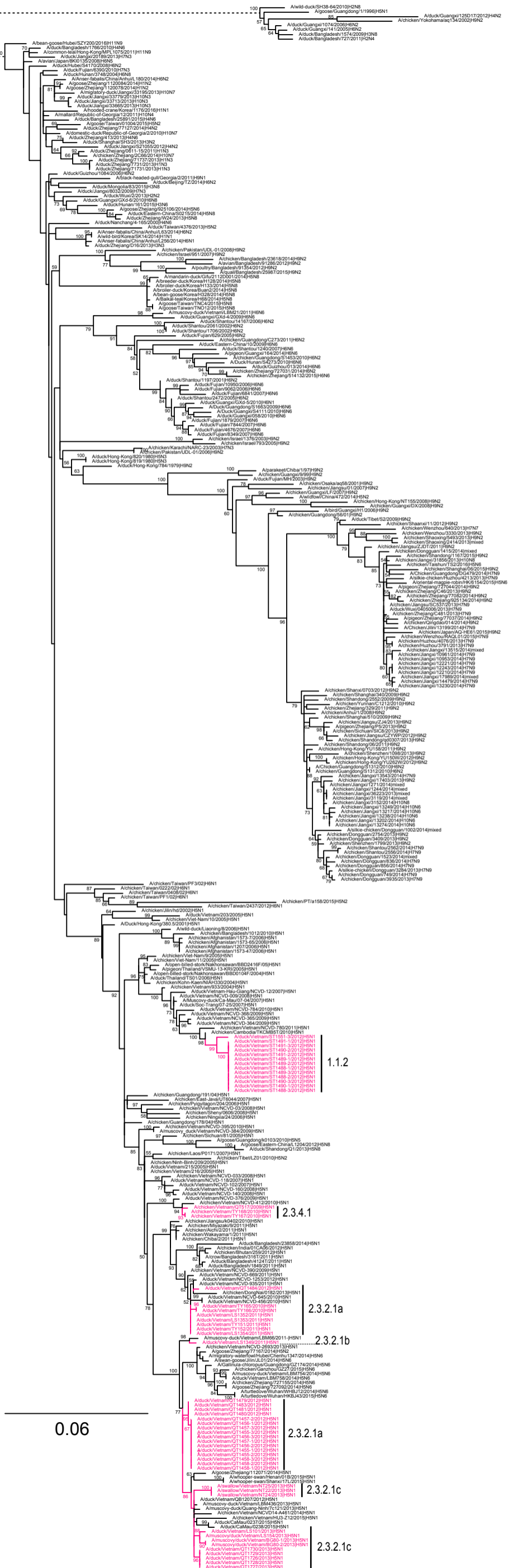

Supplement: FIGURE S7 — ML phylogeny of avian NS genes. Red font denotes H5N1 influenza viruses collected in Vietnam in this study. Bootstrap values greater than 50% are indicated at the nodes. The scale bar represents nucleotide substitutions per site. Vertical bars indicate (sub)clades. [file Data_Sheet_7.PDF]
